# Decrease in medial meniscal extrusion after physical therapy to improve knee pain and range of motion in patients with knee osteoarthritis: A retrospective study

**DOI:** 10.1371/journal.pone.0277628

**Published:** 2022-11-30

**Authors:** Hisayoshi Yoshizuka, Takanori Taniguchi, Kensuke Fukuta, Tsubasa Mitsutake, Shigenobu Honda

**Affiliations:** 1 Department of Physical Therapy, Fukuoka International University of Health and Welfare, Fukuoka City, Fukuoka, Japan; 2 Department of Rehabilitation Medicine, Honda Orthopedic Clinic, Saga City, Saga, Japan; 3 Department of Orthopedic Surgery, Honda Orthopedic Clinic, Saga City, Saga, Japan; Monash University, AUSTRALIA

## Abstract

**Background:**

Medial meniscal extrusion (MME) is the medial displacement of the meniscus, which extends beyond the tibial margin. Studies have shown an association between MME and knee pain and that surgical treatment can reduce the extent of MME. Here, we describe the beneficial effects of physical therapy as a feasible conservative treatment for MME.

**Methods:**

Data of 30 patients with knee osteoarthritis who underwent stretching of the semimembranosus tendon and passive range of motion (ROM) exercises twice a week for 8 weeks were retrospectively analyzed. MME was the measured distance between the medial meniscus and the line connecting the medial borders of the femur and tibia using ultrasound. Ultrasound findings of surrounding tissues, including the deep posterior bundle of the medial collateral ligament (dMCL), were recorded. Additionally, knee extension ROM was measured, and inner knee pain when walking was evaluated using a numerical rating scale.

**Results:**

There were significant improvements between the baseline and 8 weeks for MME in the non-weight-bearing position (3.6 ± 0.3 mm *vs*. 3.0 ± 0.4 mm), MME in the weight-bearing position (4.3 ± 0.4 mm *vs*. 3.8 ± 0.5 mm), ROM (−12.3° ± 4.1° *vs*. −3.1° ± 3.8°), and knee pain (7.0 ± 0.9 *vs*. 1.1 ± 1.4) (each *p* < 0.001). In almost all cases in which the knee extension ROM improved, the dMCL was bulging at the baseline; after 8 weeks, the dMCL was flattened, suggesting ligament tension on ultrasound imaging.

**Conclusion:**

Stretching of the semimembranosus tendon and passive ROM exercises may reduce the extent of MME in patients with knee osteoarthritis. The ultrasound findings suggest that improvement in knee extension ROM may have led to the re-acquisition of MCL tension, which may have influenced MME reduction. Therefore, physical therapy may be a feasible conservative treatment for the reduction of MME.

## Introduction

Knee osteoarthritis (KOA) is a common degenerative disease characterized by cartilage degeneration, exfoliation, and subchondral bone hyperplasia. In 2017, approximately 12.9 million patients were newly diagnosed with KOA worldwide, bringing the number of affected individuals to approximately 263 million [1]. Patients with KOA experience degenerative joint changes, muscle weakness, knee pain, and limited range of motion (ROM), all of which may cause difficulty in performing daily activities and participating in social events [2]. Pain and other associated symptoms may impair postural and balance control and increase the risk of falls [3]. As pain is often considered a dominant factor affecting the quality of life, pain relief plays an important role in the management of KOA [4].

The meniscus is a crescent-shaped fibrocartilaginous structure that plays a crucial role in load bearing and load transmission within the knee joint. Menisci actively increase the load bearing contact area by compensating for the incongruency of the articular surfaces of the tibia and femur, resulting in decreased tibiofemoral contact pressure during knee joint movements [5]. Medial meniscal extrusion (MME) is defined as the medial displacement of the body of the meniscus beyond the outermost margin of the medial tibial plateau [6]. MME can disturb the mechanical capacity of the meniscus, increase load stress, and decrease the contact area on the surface of the medial compartment of the knee [7]; therefore, it is not surprising that an association between MME and knee pain has been reported [8].

Guidelines related to the management of KOA recommend exercise therapy as a core treatment [2]. Exercise therapy has significant benefits for physical and physiological functions in KOA because exercise therapy, such as muscle strengthening and ROM exercises, promotes joint mobility, stability, balance, and cardiorespiratory function [9]. In addition, a recent systematic review and accompanying meta-analysis showed that stretching exercises resulted in significant pain reduction in individuals with KOA [2]. As for MME, past studies have revealed that surgical treatment could reduce the extent of MME [10, 11]. However, to the best of our knowledge, a feasible conservative treatment for MME, including physical therapy, has not yet been reported. The association between MME and changes in knee pain or ROM resulting from conservative therapy remains unclear.

The purpose of this study was to use the data from our own treatment to quantify the MME variation over time using ultrasound and to reveal its association with changes in knee pain and ROM resulting from physical therapy in patients with KOA.

## Materials and methods

### Study design

This was a single-center retrospective study. The study protocol followed the tenets of the Declaration of Helsinki and was approved by the Ethics Committee of the Fukuoka International University of Health and Welfare, Fukuoka, Japan (authorization number 20-fiuhw-005). Written informed consent was obtained from all study participants. All patient information was anonymized prior to the analysis.

### Study patients

We retrospectively reviewed the records of all outpatients with KOA at the Honda Orthopedic Clinic (Saga, Japan) between September 1, 2019, and March 31, 2020.

The inclusion criteria were as follows: (1) diagnosis of medial KOA; (2) MME greater than 3 mm confirmed by ultrasound; (3) limited ROM on knee joint extension; (4) inner knee pain when walking; (5) undergoing continuous physical therapy twice a week for 8 weeks (8w); and (6) continuous ultrasound examination and evaluation of ROM and pain every 2 weeks (2w) for 8w. The exclusion criteria were as follows: (1) bilateral KOA; (2) meniscal injury and previous meniscal surgery; (3) medial collateral ligament (MCL) injury; (4) osteophytes around the joint space of the knee joint; (5) hydrarthrosis of the knee; (6) presence of neurological, inflammatory, or systemic disease; (7) missing measurements; and (8) loss to follow-up.

Although patients were assigned to the physical therapist at the discretion of the treating physician, physical therapy for inner knee pain and ROM was implemented, as described below, as the standard care regime at our clinic. The patients who declined participation in the study also received the same care as those who agreed to participate.

Finally, this retrospective study evaluated 30 knee joints of 30 patients (Table 1). The cohort consisted of 5 men and 25 women, with ages ranging from 60 to 90 years. The patients’ relevant past medical histories were as follows: high blood pressure in seven patients, diabetes mellitus in two patients, high tibial osteotomy in one patient, tibial diaphyseal fracture in one patient, and lumbar compression fracture in one patient.

**Table 1:** Baseline characteristics of patients with knee osteoarthritis.

Variable	Overall (n = 30)
**Age, years**	70 ± 9 (60–90)
**Sex**	
**Men, n (%)**	5 (16.7%)
**Women, n (%)**	25 (83.3%)
**K&L grade, n (%)**	
**Grade 1**	5 (16.7%)
**Grade 2**	9 (30.0%)
**Grade 3**	11 (36.7%)
**Grade 4**	5 (16.7%)
**FTA, degrees**	180.0 ± 3.6 (175.3–188.1)

Values are expressed as mean ± standard deviation (range) or number (percentage).

Abbreviations: K&L grade: Kellgren–Lawrence grade; FTA: femorotibial angle.

### Physical therapy for the knee joint

All outpatients received physical therapy for the knee joint conducted by a single experienced physical therapist (K.F.) twice a week for 8w. Each treatment session lasted 20 min and consisted of stretching and passive ROM exercises to improve the ROM and inner knee pain. As the direct arm of the semimembranosus tendon was tender to palpation in all patients, the physical therapist manually stretched the direct arm and passively extended and internally rotated the knee joint.

### Measuring methods

**Fig 1:**
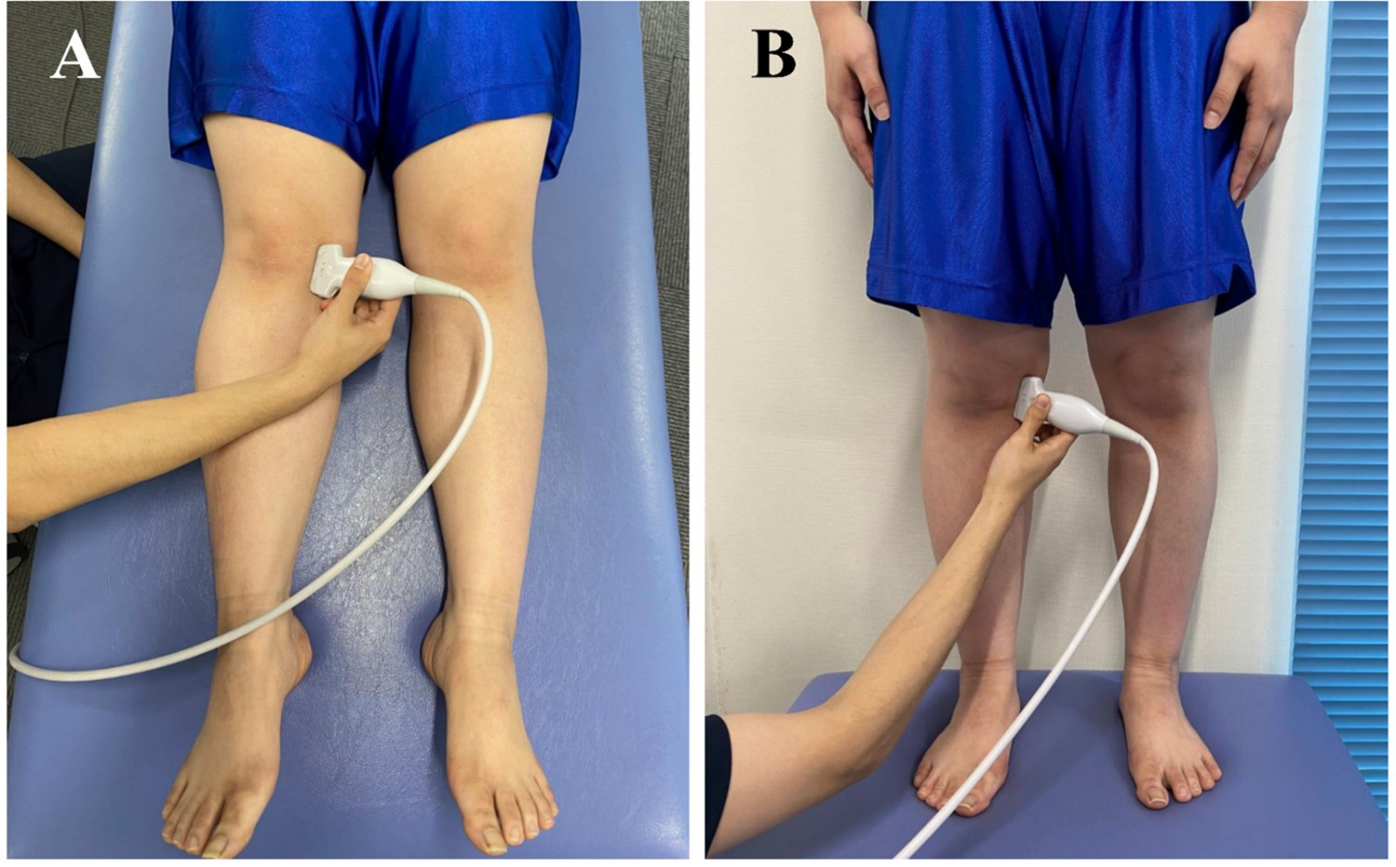
Measurement of the medial knee joint using ultrasound. Ultrasound assessed the medial meniscus when the patient was in the supine (**A**) and standing (**B**) positions. Both measurements were performed at maximum knee joint extension in each patient. The transducer was placed longitudinally at the level of the medial collateral ligament at the medial aspect of the knee joint.

**Fig 2:**
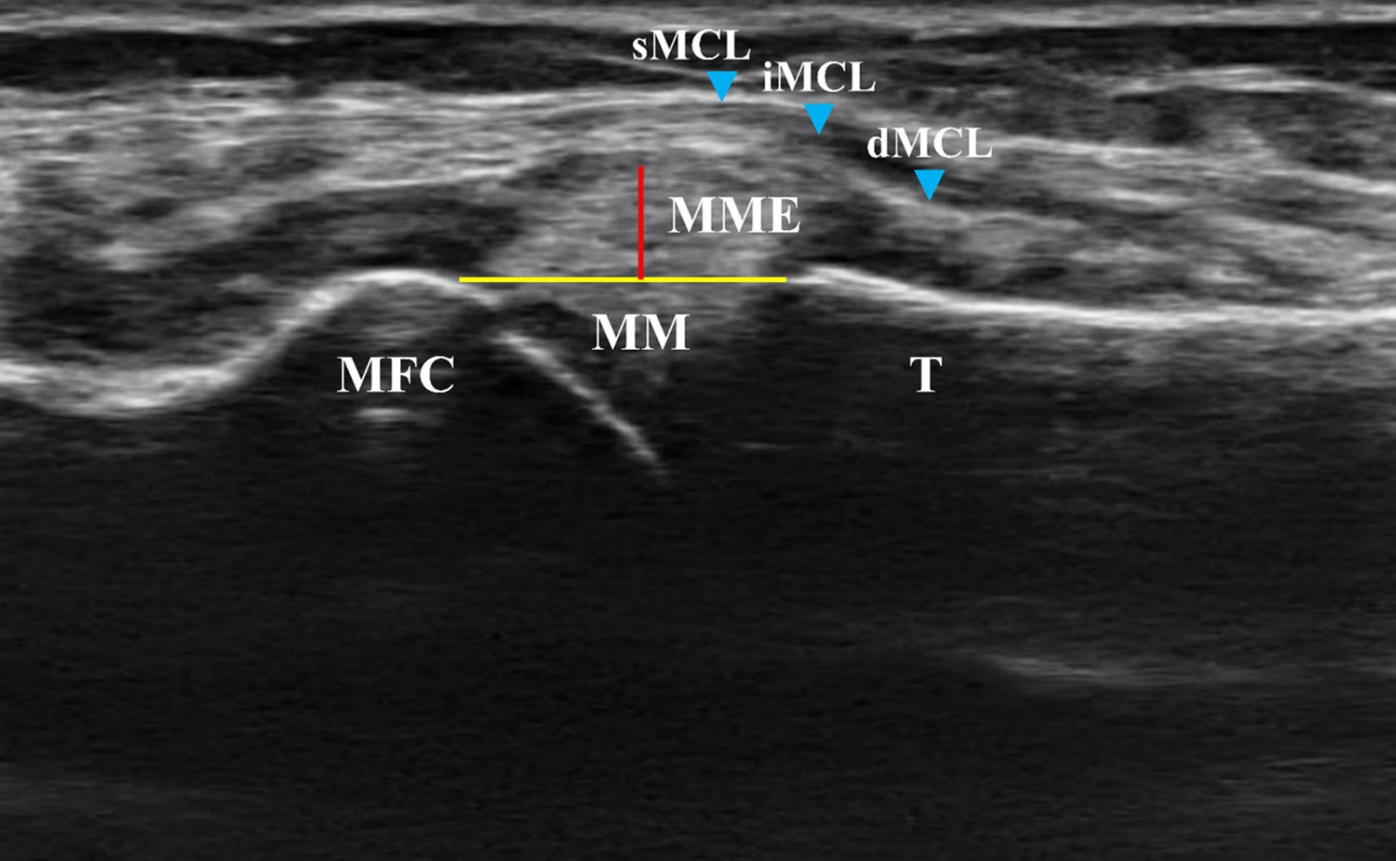
Definition of MME measurements. Longitudinal ultrasound images at the level of the MCL at the medial aspect of the knee joint. MME (red line) was measured as the distance between the outermost border of the medial meniscus and the line connecting the medial borders of the femur and tibia (yellow line). Abbreviations: sMCL (light blue arrowhead), superficial bundle of the medial collateral ligament (MCL); iMCL (light blue arrowhead), intermediate superficial bundle of the MCL; dMCL (light blue arrowhead), deep posterior bundle of the MCL; MME, medial meniscal extrusion; MM, medial meniscus; MFC, medial femoral condyle; T, tibia.

According to Özdemir and Turan [12], MME was evaluated in the non-weight-bearing (NWMME) and weight-bearing (WMME) positions using ultrasound (SONIMAGE HS2, Konica Minolta Japan, Inc., Tokyo, Japan) (Fig 1). The MME value was defined as the distance between the outermost border of the medial meniscus and the line connecting the medial borders of the femur and tibia (Fig 2). The minimum unit of recording was 0.01 mm. Ultrasound findings of the surrounding tissues, including the MCL, were also confirmed (Fig 2).

Inner knee pain during walking was assessed using the numerical rating scale (NRS), which measured pain from 0 (no pain) to 10 (maximum pain imaginable). Knee extension ROM was measured in the supine position using a goniometer, and the minimum unit of recording was 1°.

All measurements were performed by a single well-trained physical therapist (K.F.) who had 7 years of experience in performing ultrasound. Repeated measurements were used to determine the changes in MME, ROM, and pain at five time periods: before intervention (baseline), 2w, 4 weeks (4w), 6 weeks (6w), and 8w. The accuracy of the technique was confirmed by repeating the procedure for a total of three times.

### Statistical analyses

All statistical analyses were conducted using SPSS Statistics version 26 (IBM Corporation, Armonk, NY, USA), and the level of statistical significance was set at a *P* value of 0.05.

The normality distribution of the quantitative data was determined using the Shapiro–Wilk test. Mauchly’s sphericity test was performed to determine whether a violation of sphericity had occurred. Temporal changes in the parameters were assessed using the Greenhouse–Geisser correction method to correct the distortion or results of the Friedman test. Tukey’s test or Bonferroni correction test was used to make post-hoc pairwise comparisons. To evaluate the effects of the intervention, the changes in all parameters were calculated by subtracting the values at baseline from each value at 2w, 4w, 6w, and 8w. Spearman’s rank correlation coefficient was calculated to investigate the relationship between the changes in all parameters.

## Results

**Fig 3:**
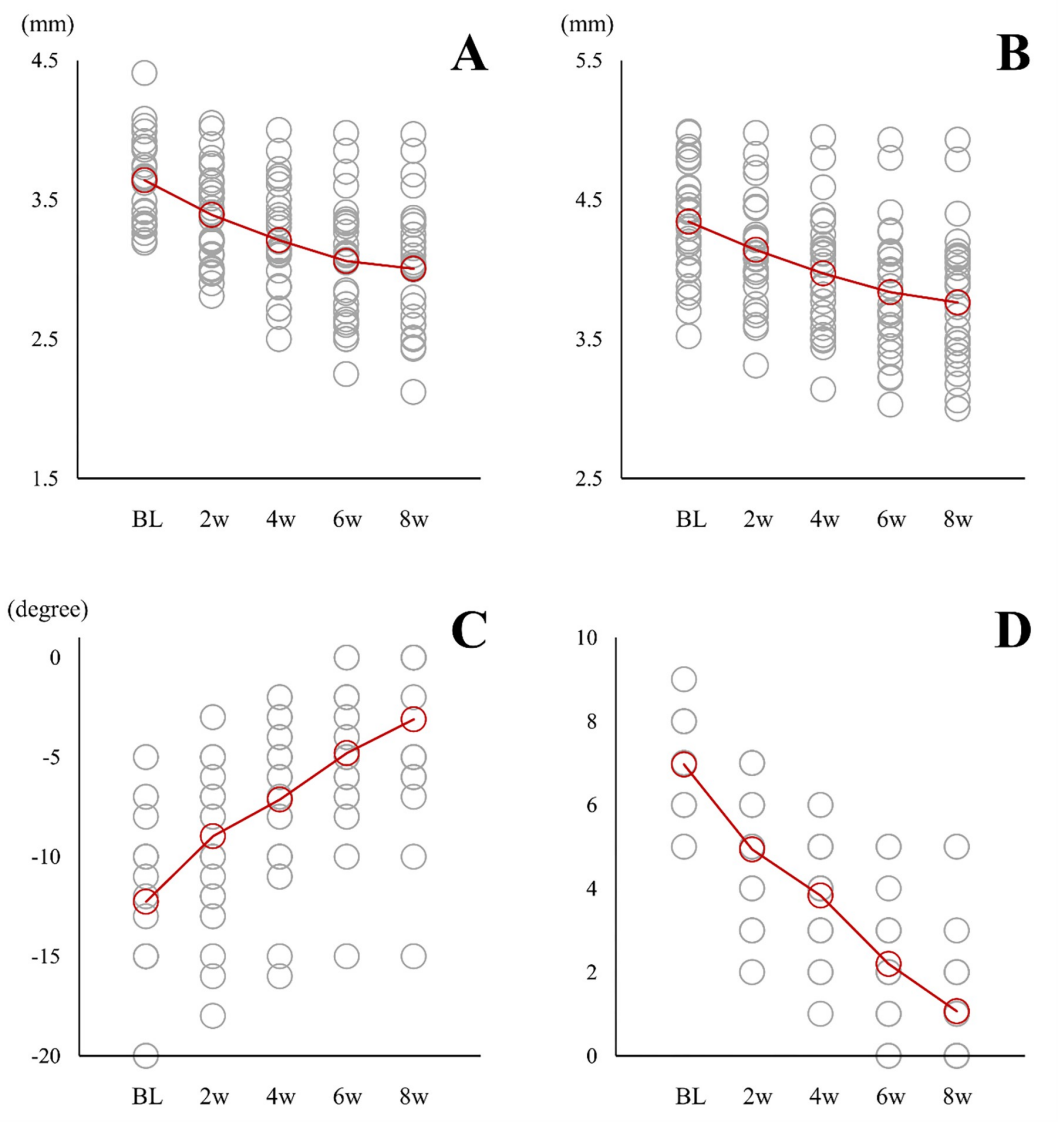
Temporal changes in the parameters. Each scatterplot indicates non-weight-bearing medial meniscal extrusion (**A**), weight-bearing medial meniscal extrusion (**B**), range of motion (**C**), and knee pain according to the numerical rating scale (**D**). Red and gray circles show mean values and values in each patient, respectively. Abbreviations: BL, baseline.

The temporal changes in the parameters are presented in Fig 3, and the results of the statistical analyses of these data are summarized in Table 2. All parameters showed significance in NWMME (*F* [4, 116] = 77.265, *p* < 0.001), WMME (*F* [4, 116] = 132.783, *p* < 0.001), ROM (*p* < 0.001), and pain (*p* < 0.001). Post-hoc Tukey’s tests revealed that NWMME and WMME showed significant differences between the respective periods (*p* < 0.01 or *p* < 0.001), except between 6w and 8w. NWMME was significantly lower at 8w (3.0 ± 0.4 mm, 95% confidence interval [CI]: 2.8 to 3.2 mm) than at baseline (3.6 ± 0.3 mm, 95% CI: 3.5 to 3.8 mm) (*p* < 0.001). WMME was also significantly lower at 8w (3.8 ± 0.5 mm, 95% CI: 3.6 to 3.9 mm) than at baseline (4.3 ± 0.4 mm, 95% CI: 4.2 to 4.5 mm) (*p* < 0.001). Post-hoc Bonferroni correction tests revealed that the ROM and pain showed significant differences at 4w, 6w, and 8w (*p* < 0.05 or *p* < 0.001). ROM was significantly greater at 8w (−3.1° ± 3.8°, 95% CI: −4.5° to −1.7°) than at baseline (−12.3° ± 4.1°, 95% CI: −13.8° to −10.7°) (*p* < 0.001). Pain was significantly lower at 8w (1.1 ± 1.4, 95% CI: 0.5 to 1.6) than at baseline (7.0 ± 0.9, 95% CI: 6.6 to 7.3) (*p* < 0.001). The change in each parameter from baseline to any period is shown in Table 3. The mean differences (95% CI) between baseline and 8w reached −0.6 ± 0.4 mm (−0.8 to −0.5 mm) for NWMME, −0.6 ± 0.2 mm (−0.7 to −0.5 mm) for WMME, 9.2° ± 2.8° (8.1° to 10.2°) for ROM, and −5.9 ± 1.5 (−6.4 to −5.4) for pain. At 8w, tenderness of the direct arm of the semimembranosus tendon disappeared in all patients.

**Table 2:** Statistical analyses of temporal changes of the parameters.

	Baseline	2 weeks	4 weeks	6 weeks	8 weeks	*P* value
**NWMME, mm**	3.6 ± 0.3 (3.5 to 3.8)	**3.4 ± 0.3**^a^ (3.3 to 3.5)	**3.2 ± 0.4**^a^^,^^b^ (3.1 to 3.4)	**3.1 ± 0.4**^a^^,^^b^^,d^ (2.9 to 3.2)	**3.0 ± 0.4**^a^^,^^b^^,^^c^ (2.8 to 3.2)	**< 0.001**
**WMME, mm**	4.3 ± 0.4 (4.2 to 4.5)	**4.1 ± 0.4**^a^ (4.0 to 4.3)	**4.0 ± 0.4**^a^^,^^b^ (3.8 to 4.1)	**3.8 ± 0.4**^a^^,^^b^^,^^c^ (3.7 to 4.0)	**3.8 ± 0.5**^a^^,^^b^^,^^c^ (3.6 to 3.9)	**< 0.001**
**ROM extension, degrees**	−12.3 ± 4.1 (−13.8 to −10.7)	−9.0 ± 3.8 (−10.4 to −7.5)	**−7.1 ± 3.6**^a^ (−8.5 to −5.8)	**−4.8 ± 3.4**^a^^,^^b^ (−6.1 to −3.5)	**−3.1 ± 3.8**^a^^,^^b^^,^^c^ (−4.5 to −1.7)	**< 0.001**
**Knee pain NRS**	7.0 ± 0.9 (6.6 to 7.3)	4.9 ± 1.5 (4.4 to 5.5)	**3.8 ± 1.3**^a^ (3.4 to 4.3)	**2.2 ± 1.4**^a^^,^^b^^,e^ (1.7 to 2.7)	**1.1 ± 1.4**^a^^,^^b^^,^^c^ (0.5 to 1.6)	**< 0.001**

Significant values are indicated in bold. The values are presented as the mean ± standard deviation (95% confidence interval).

Abbreviations: NWMME, non-weight-bearing medial meniscal extrusion; WMME, weight-bearing medial meniscal extrusion; ROM, range of motion; NRS, numerical rating scale.

^a^ Indicates significant difference from baseline (*p* < 0.001).

^b^ Indicates significant difference from 2 weeks (*p* < 0.001).

^c^ Indicates significant difference from 4 weeks (*p* < 0.001).

^d^ Indicates significant difference from 4 weeks (*p* < 0.01).

^e^ Indicates significant difference from 4 weeks (*p* < 0.05).

**Table 3:** Amount of change in each parameter from baseline.

	Δ 2w−BL	Δ 4w−BL	Δ 6w−BL	Δ 8w−BL
**NWMME, mm**	−0.2 ± 0.2 (−0.3 to −0.2)	−0.4 ± 0.3 (−0.5 to −0.3)	−0.6 ± 0.3 (−0.7 to −0.5)	−0.6 ± 0.4 (−0.8 to −0.5)
**WMME, mm**	−0.2 ± 0.2 (−0.3 to −0.1)	−0.4 ± 0.2 (−0.4 to −0.3)	−0.5 ± 0.2 (−0.6 to −0.4)	−0.6 ± 0.2 (−0.7 to −0.5)
**ROM extension, degrees**	3.3 ± 1.4 (2.8 to 3.8)	5.1 ± 1.8 (4.5 to 5.8)	7.5 ± 1.9 (6.8 to 8.2)	9.2 ± 2.8 (8.1 to 10.2)
**Knee pain NRS**	−2.0 ± 1.3 (−2.5 to −1.6)	−3.1 ± 1.1 (−3.5 to −2.7)	−4.8 ± 1.3 (−5.3 to −4.3)	−5.9 ± 1.5 (−6.4 to −5.4)

The difference (**Δ)** was calculated by subtracting the baseline values from each value at the four time periods. The values are presented as the mean ± standard deviation (95% confidence interval).

Abbreviations: BL, baseline; NWMME, non-weight bearing medial meniscal extrusion; WMME, weight bearing medial meniscal extrusion; ROM, range of motion; NRS, numerical rating scale

**Fig 4:**
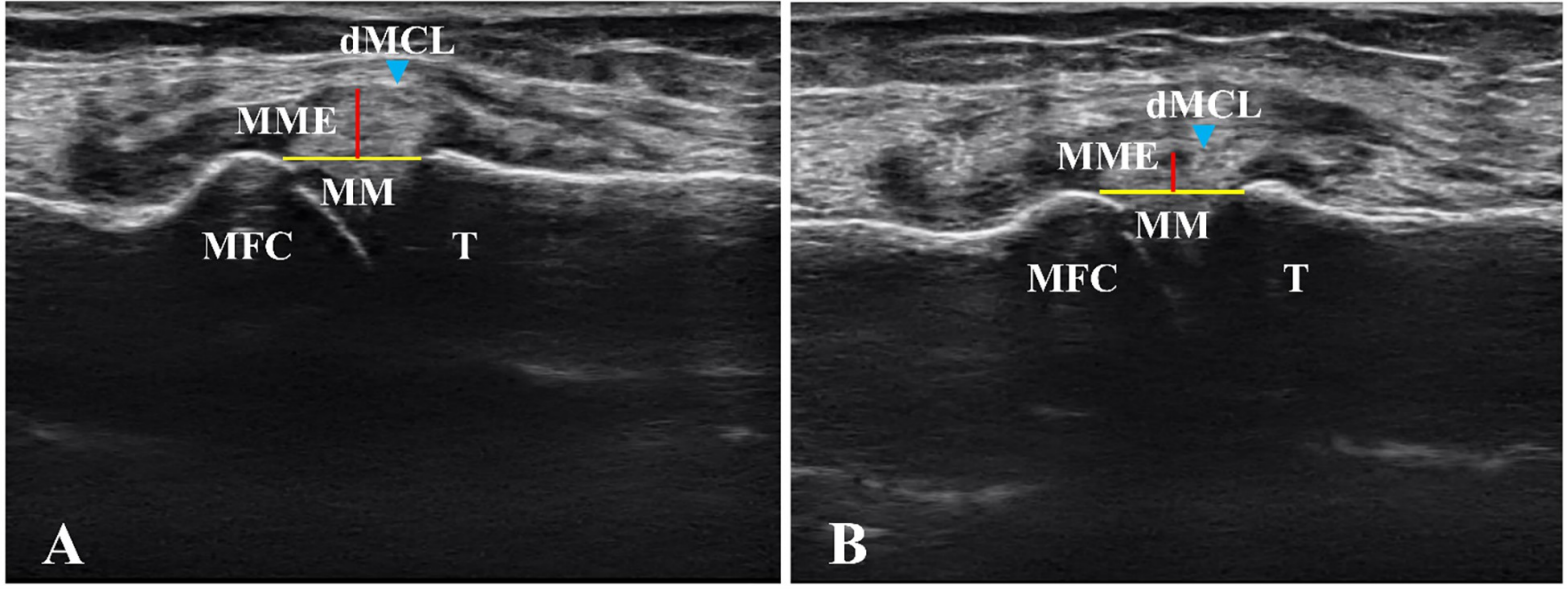
Images from a subject showing changes in MME at baseline (A) and 8 weeks (B). MME (red line) was lower at 8 weeks than at baseline. The dMCL (light blue arrowhead) was bulging at baseline and flattened at 8 weeks. Abbreviations: dMCL, deep posterior bundle of the medial collateral ligament; MM (yellow line), medial meniscus; MME; medial meniscus extrusion; MFC, medial femoral condyle; T, tibia.

The findings from one patient, which illustrate the changes in ultrasound images of the knee joint from baseline to 8w, are presented in Fig 4. On ultrasound imaging, the deep MCL (dMCL) was bulging at baseline and flattened at 8w. These findings were observed in almost all cases in which the ROM of knee extension improved.

**Fig 5:**
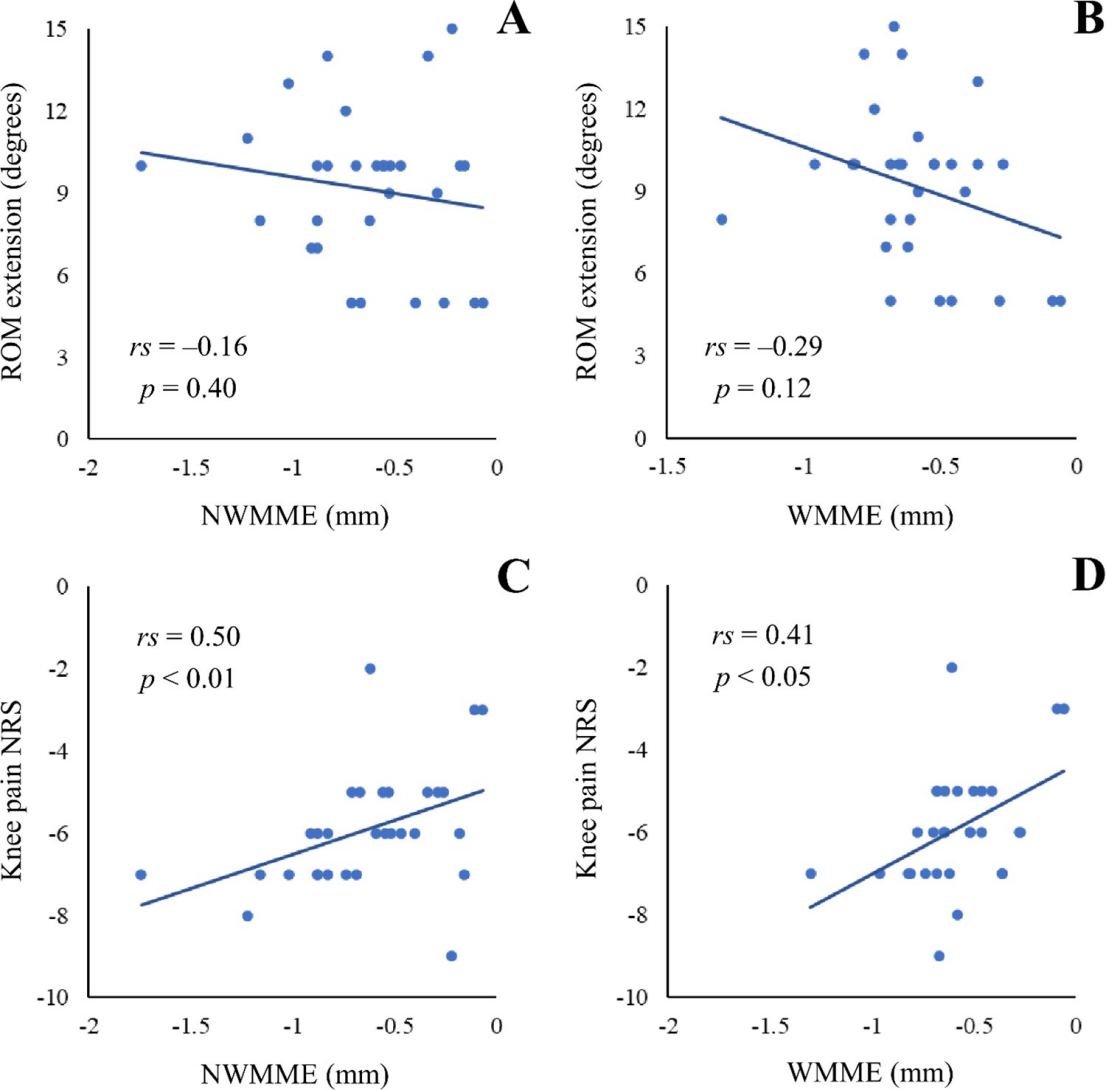
Scatter plot of changes in the parameters from baseline to 8 weeks. Between NWMME and ROM (A), WMME and ROM (B), NWMME and pain score (C), and WMME and pain score (D). All approximately straight lines are shown as the eye guide. Abbreviations: ROM, range of motion; NWMME, non-weight-bearing medial meniscal extrusion; WMME, weight-bearing medial meniscal extrusion; NRS, numerical rating scale; *rs*, Spearman’s rank correlation coefficient; *p*, *p* value.

To confirm the relationship among the changes in the parameters, scatter plot diagrams were prepared, and correlation coefficients were calculated (Fig 5). Moderate positive correlations were observed between NWMME and pain (*rs* = 0.50, *p* < 0.01) and between WMME and pain (*rs* = 0.41, *p* < 0.05). However, there was no significant correlation between ROM and MME.

## Discussion

The most important finding of the present study was that physical therapy may reduce the extent of MME in patients with KOA. A conservative treatment for MME has not been reported, and we believe that this is the first study to examine the relationship between changes in MME and physical therapy aimed at improving knee pain and ROM limitation. Our results showed significant improvements in NWMME, WMME, ROM, and knee pain (Table 2). Thus, in patients with KOA who experience knee pain and limited extension ROM, it is suggested that physical therapy may not only improve ROM and pain but also reduce MME. As therapy consisted only of stretching and passive ROM exercises, non-use of equipment or special techniques during therapy is an attractive alternative in KOA management.

The amount of change in knee extension ROM at 8w reached 9.2° ± 2.8° (95% CI: 8.1° to 10.2°) (Table 3). In this study, to improve knee joint extension ROM limitation and inner knee pain, a physical therapist applied intervention on the direct arm of the semimembranosus tendon [13] where there was tenderness. As the semimembranosus muscle is a knee flexor, the decreased extensibility of this tendon is a limiting factor in extension ROM. The direct arm attaches to the posterior aspect of the coronary ligament of the posterior horn of the medial meniscus [14], and the improvement in direct arm extensibility may affect not only the extension ROM but also the extent of MME. However, the normal medial meniscus moves laterally with knee flexion and medially with extension [15, 16]. In addition, MME is increased during extension rather than during flexion [16]. Regarding the change from baseline to 8w in this study, no significant correlation was found between ROM and MME (Fig 5). Therefore, although stretching exercises may have directly improved extension ROM, it is unlikely that the improvement in extension ROM had a direct effect on MME reduction.

Based on the ultrasound images in this study, the MCL, which is found medially in the knee joint, may be a factor in MME reduction. The ultrasound images at 8w (Fig 4B) showed flattening of the dMCL, suggesting ligament tension. In almost all patients in whom ROM knee extension improved, these findings were not present at baseline and only appeared during treatment. A previous study reported that ultrasound findings in 55 of 90 symptomatic knees included protrusion of MME associated with displacement of the MCL [17]. In addition, Gale et al. described that for the meniscus to translocate, several anatomical conditions must occur, including laxity in the MCL and possibly other meniscal attachments [18]. Since the MCL relaxes during knee flexion and tenses during knee extension, it does not achieve full tension when there is limited extension ROM. Although the average extension ROM was −12.3° ± 4.1° at baseline with all patients having more than 5° of restriction, it improved to −3.1° ± 3.8° at 8w (*p* < 0.001) (Table 2). As the dMCL is a thickening of the medial joint capsule with meniscotibial and meniscofemoral attachments [19], the re-acquisition of MCL tension with improved extension ROM may have affected the decrease in MME.

Inner knee pain when walking improved from a mean of 7.0 ± 0.9 at baseline to 1.1 ± 1.4 at 8w (*p* < 0.001) (Table 2). Patients with KOA may respond to the pain with muscle tension and tend to avoid physical activity in an attempt to prevent the pain [2]. Consequently, the connective tissue around the joint becomes fibrotic owing to immobility or inactivity [20]. Additionally, adaptive shortening of muscles and capsular adherence can also be implicated in the pain [2]. Stretching exercises could be effective for alleviating pain because they can cause relaxation of the muscle fibres, thereby increasing muscle length and improving muscle tone [21]. Therefore, stretching exercises might have directly alleviated the inner knee pain in our patients.

Regarding the change from baseline to 8w, there was a moderate positive correlation between NWMME and pain (*rs* = 0.50, *p* < 0.01) and between WMME and pain (*rs* = 0.41, *p* < 0.05) (Fig 5). The association between MME and knee pain has previously been reported [8] and is supported by the present results. The meniscus is a crucial mechanical component of the knee and is responsible for load transmission and shock absorption; it transmits 40%–60% of the load in a standing position and 90% during knee flexion [22]. Therefore, the decrease in MME may also have influenced the reduction in inner knee pain during walking.

The results of post-hoc analyses also suggested a desirable duration for the intervention for MME. NWMME and WMME were significantly different between the respective periods (*p* < 0.01 or *p* < 0.001), except between 6w and 8w (Table 2). Thus, physical therapy as performed in this study is expected to be effective up to 6w, with a plateau after 6w.

There is still no consensus regarding the extent of MME that can be considered physiological; however, some authors have accepted up to 3 mm of MME as normal [23]. Our results at 8w showed that the NWMME decreased to 3 mm, whereas the WMME remained above 3 mm. In addition, malalignment and obesity have been reported to be associated with a higher risk of MME [24]. Considering multiple factors, further studies regarding the effectiveness of conservative therapy for MME are required.

### Limitations

This study had some limitations. First, as the number of patients per Kellgren–Lawrence grade varied, the effect of grade on the change in MME could not be evaluated. Second, as MME was measured at maximum knee extension in each patient, the joint angle of measurement could not be standardized. However, despite the MME being greater during extension than during flexion [16], the present results showed a significant difference in MME; however, further comparative studies in the same position are required. Third, although the accuracy of the technique was confirmed by repeating the procedure for a total of three times, all measurements were performed by a single researcher. The inter- and intra-rater reliabilities of the measurement were not tested. Fourth, our data were not compared with a control group. Fifth, the specific impact of the MCL and direct arm of the semimembranosus on the MME is unclear. Therefore, further imaging and anatomical studies and a randomized controlled trial are warranted.

## Conclusions

Our results revealed that physical therapy may reduce the extent of MME in patients with KOA. The ultrasound findings suggest that the improvement in extension ROM may have led to re-acquisition of MCL tension, which may have influenced MME reduction. Physical therapy may be a feasible conservative treatment for the reduction of MME.

## Supporting information

S1 Data (XLSX)
